# Morphologic and genetic characterization of *Pterygodermatites* (*Mesopectines*) *valladaresi* n. sp. (Nematoda, Rictulariidae), a parasite of the mouse *Mus musculus* (Rodentia, Muridae) from the Canary Islands (Spain)[Fn FN1]

**DOI:** 10.1051/parasite/2022057

**Published:** 2022-12-21

**Authors:** Jordi Miquel, Natalia Martín-Carrillo, Alexis Ribas, Santiago Sánchez-Vicente, Carlos Feliu, Pilar Foronda

**Affiliations:** 1 Secció de Parasitologia, Departament de Biologia, Sanitat i Medi Ambient, Facultat de Farmàcia i Ciències de l’Alimentació, Universitat de Barcelona, Avgda. Joan XXIII, sn 08028 Barcelona Spain; 2 Institut de Recerca de la Biodiversitat (IRBio), Universitat de Barcelona Avgda. Diagonal, 645 08028 Barcelona Spain; 3 Instituto Universitario de Enfermedades Tropicales y Salud Pública de Canarias, Universidad de La Laguna, Avda. Astrofísico F. Sánchez, sn 38203 La Laguna Canary Islands Spain; 4 Center for Infection and Immunity, Mailman School of Public Health, Columbia University 722 West 168th Street New York NY 10032 USA; 5 Departamento Obstetricia y Ginecología, Pediatría, Medicina Preventiva y Salud Pública, Toxicología, Medicina Legal y Forense y Parasitología, Facultad de Farmacia, Universidad de La Laguna, Avda. Astrofísico F. Sánchez, sn 38203 La Laguna Canary Islands Spain

**Keywords:** *Pterygodermatites* (*Mesopectines*) *valladaresi* n. sp., Rictulariidae, *Mus musculus*, Muridae, Canary Islands

## Abstract

A new rictulariid nematode *Pterygodermatites* (*Mesopectines*) *valladaresi* n. sp., parasite of the house mouse *Mus musculus* (Rodentia: Muridae) in the Canary Islands (Spain) is described by means of light and scanning electron microscopy. The new species belongs to the subgenus *Mesopectines* characterized by a more or less dorsal orientation of the buccal capsule, the presence of three oesophageal teeth, the morphology of the oral denticles and the Spirurida type of arrangement of caudal papillae in males. The most discriminant characteristics between the new species and the existing species in the subgenus *Mesopectines* are (a) the number of cuticular projection pairs (62–64), (b) the size of right and left spicules (respectively, 62–90 µm and 123–139 µm), (c) the number of midventral fans in males (3–4), (d) the number of prevulvar/total cuticular projection pairs (38–42/63–71), (e) the posterior differentiation of combs into spines in relation to the position of the vulva and (f) the anterior position of the vulva in relation to the oesophagus-intestine junction in females. Parasitized hosts and geographical distribution are also useful criteria to distinguish *P.* (*Me.*) *valladaresi* n. sp. from the remaining species of the subgenus. In addition, the *cox1* sequence of the new species is provided and compared with available data of related species.

## Abstract (fr)

Un nouveau nématode rictulaire, P*terygodermatites (Mesopectines) valladaresi* n. sp., parasite de la souris domestique *Mus musculus* (Rodentia, Muridae) aux îles Canaries (Espagne) est décrit par microscopie optique et électronique à balayage. La nouvelle espèce appartient au sous-genre *Mesopectines* caractérisé par une orientation plus ou moins dorsale de la capsule buccale, la présence de trois dents œsophagiennes, la morphologie des denticules buccaux et le type Spirurida d'arrangement des papilles caudales chez les mâles. Les caractéristiques les plus discriminantes entre la nouvelle espèce et les espèces existantes du sous-genre *Mesopectines* sont (a) le nombre de paires de projections cuticulaires (62–64), (b) la taille des spicules droit et gauche (respectivement, 62–90 µm et 123–139 µm), (c) le nombre de bourrelets cuticulaires médio-ventraux chez les mâles (3–4), (d) le nombre de peignes prévulvaires par rapport au nombre total de peignes (38–42/63–71), (e) la transformation en épines des peignes après la vulve et (f) la position antérieure de la vulve par rapport à la jonction œsophage-intestin chez les femelles. Les hôtes parasités et la répartition géographique sont également des critères utiles pour distinguer *P. (Me.) valladaresi* n. sp. des autres espèces du sous-genre. De plus, la séquence du *cox1* de la nouvelle espèce est fournie et comparée aux données disponibles sur les espèces apparentées.

## Introduction

The genus *Pterygodermatites* Wedl, 1861 includes 68 rictulariid nematode species parasitizing a wide range of mammalian vertebrates [41]. This genus has five subgenera, namely *P.* (*Mesopectines*) Quentin, 1969, *P.* (*Multipectines*) Quentin, 1969, *P.* (*Neopaucipectines*) Quentin, 1969, *P.* (*Paucipectines*) Quentin, 1969 and *P.* (*Pterygodermatites*) Quentin, 1969 [6, 31]. Species of these subgenera are mainly characterized by the orientation of the buccal capsule, the morphology of oral denticles and other peribuccal structures, the morphology of oesophageal teeth, the number of cuticular projection pairs (combs and spines), the size of spicules, and the type and arrangement of cloacal papillae. The geographical distribution of *Pterygodermatites* spp. is wide and it is also a feature that can be used to differentiate some of the species [6, 31].

Recently, Simões et al. [41] compiled all the available literature providing the checklist of species of the genus *Pterygodermatites*. According to these authors, the subgenera *P.* (*Paucipectines*) and *P.* (*Mesopectines*) are the most diverse of the five subgenera with 27 and 25 described species, respectively. There are 16 additional species belonging to the remaining subgenera: six species of *P.* (*Multipectines*), four of *P.* (*Neopaucipectines*) and six of *P.* (*Pterygodermatites*). The subgenus *P.* (*Mesopectines*) includes species widely distributed in Africa and Asia parasitizing species of the orders Rodentia, Carnivora and Primates [31, 41].

In the present study, we describe a new species, *P.* (*Me.*) *valladaresi* n. sp., parasitizing the house mouse (*Mus musculus*) in the Canary Islands (Spain). Additionally, the sequence of the mitochondrial cytochrome c oxidase subunit I gene (MT-CO1) is provided and compared with available data of related species.

## Materials and methods

### Ethics

Animal work was approved in accordance with the Spanish Government Laws 42/2007 and RD 630/2013, the Canary Government law 151/2001 (references FYF141/10, FYF205/09, EEI-007/2019, ADL/mjb, MRR/rsh, A/EST-030/2016, AFF115/16 and EEI-007/2019), and the Ethic Committees of Research and Animal Welfare of Universidad de La Laguna (Protocol number CEIBA2018-0330).

### Specimens

**Figure 1:**
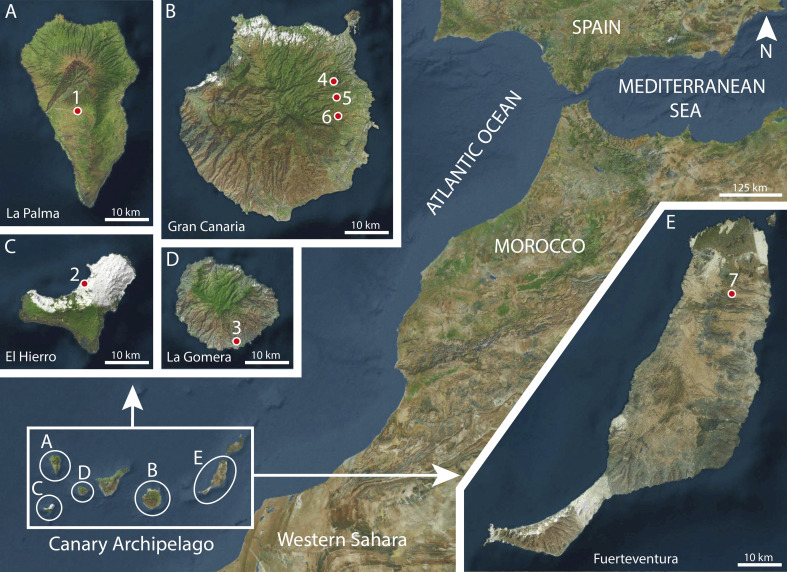
Localities where *Mus musculus* parasitized by *Pterygodermatites* (*Mesopectines*) *valladaresi* n. sp. were captured on the islands of La Palma, El Hierro, La Gomera, Gran Canaria and Fuerteventura. 1, El Paso; 2, Lagartario-Frontera; 3, Gasolinera-Antocojo; 4, La Barrera; 5, Lomo del Conde; 6, Pasadilla; 7, La Oliva (type locality).

Between 2008 and 2012, 721 house mice, *Mus musculus* Linnaeus, 1758 (Rodentia: Muridae), were captured in the Canary Islands, namely in Fuerteventura (106), Lanzarote (137), La Graciosa (42), Gran Canaria (41), Tenerife (111), La Gomera (31), La Palma (80) and El Hierro (173) [36]. The studied rodents were captured by means of Sherman and Firobin traps, sacrificed by cervical dislocation and then scanned for intestinal helminths. The new species described here *P.* (*Me.*) *valladaresi* n. sp. was found in mice from five islands: Fuerteventura, Gran Canaria, La Gomera, La Palma and El Hierro (Fig. 1). The examination of other Muridae rodents, namely 14 *Rattus norvegicus* (Berkenhout, 1769) (Gran Canaria, 1; Tenerife, 11; La Palma, 2) and 244 *Rattus rattus* (Linnaeus, 1758) (Fuerteventura, 25; Lanzarote, 20; Gran Canaria, 19; Tenerife, 94; La Gomera, 17; La Palma, 16; El Hierro, 53) were negative for the presence of this nematode [36].

### Light microscopy study

Specimens were mounted in Amann lactophenol on slides and then observed under the light microscope. Drawings were made with the aid of a drawing tube and later modified using Adobe Illustrator software (Adobe, San José, CA, USA). All measurements are given in micrometres (except where indicated).

### Scanning electron microscopy study

Some worms (1 male and 4 females) were preserved for scanning electron microscopy (SEM) examination. Initially they were fixed in 70% ethanol in the field and later, in the laboratory, they were dehydrated in an ethanol series and critical point dried with carbon dioxide in an Emitech K850X (Quorum Technologies Ltd., Laughton, East Sussex, UK). Finally, specimens were mounted on stubs with conductive adhesive tape and colloidal silver, coated with carbon in an Emitech K950X (Quorum Technologies Ltd.) evaporator, and examined using a Field Emission SEM JSM-7001F (Jeol Ltd., Tokyo, Japan) at 10 kV in the “Centres Científics i Tecnològics” of the University of Barcelona (CCiTUB).

### Molecular analysis and phylogenetic tree

Genomic DNA samples were isolated from the mid-section fragment of *P.* (*Me.*) *valladaresi* n. sp. following López et al. [23]. The DNA extraction procedure was checked using DeNovix DS-11+ Spectrophotometer (DeNovix Inc., Wilmington, DE, USA).

DNA amplification by PCR was conducted using the primer cocktail as described by Prosser et al. [30], for the barcode region of the mitochondrial cytochrome c oxidase subunit I gene (MT-CO1). The PCR amplification contained 1× Buffer (Bioline Ltd., London, UK), 0.2 mM of each dNTP (Bioline Ltd.), 0.5 µL of each primer cocktail (10 µM of a three-forward-primers mix, and 10 µM of a three-reverse-primers mix), 1U of Taq DNA polymerase (Bioline Ltd.), 1.5 mM MgCl_2_ (Bioline Ltd.), and 20–30 ng of total genomic DNA in a total volume of 50 µL. Amplification was conducted with XP Cycler (Hangzhou Bioer Technology Co. Ltd., Hangzhou, China) using the following parameters: 94° C for 1 min; five cycles at 94 °C for 40 s, 45 °C for 40 s, 72 °C for 1 min; followed by 35 cycles at 94 °C for 40 s, 51 °C for 40 s, 72 °C for 1 min; and a final extension at 72 °C for 5 min [30]. The resulting amplifications were visualized on 1.2% agarose gel at 100 V for 45 min.

The PCR product was sequenced by Macrogen Spain Inc. (Madrid, Spain) with primers NemF1_t1 and NemR1_t1 [30]. The analysis of the sequences was carried out with software MEGA X [22], using the multiple alignment program ClustalW included in MEGA X, and minor corrections were made by hand.

**Figure 2:**
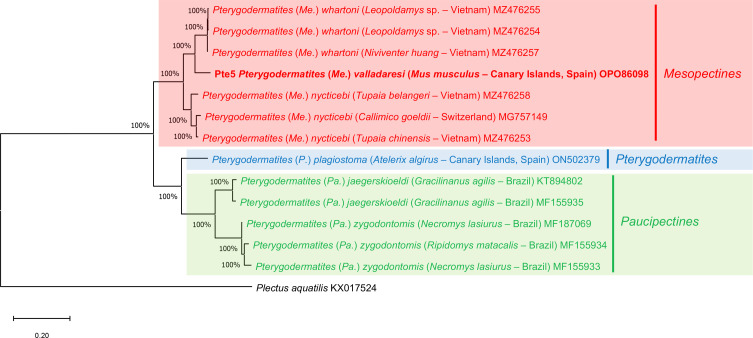
Phylogenetic analysis using the Maximum Likelihood method with p-distance and 1,000 bootstrap replications based on the MT-CO1 gene sequences exploring the relationships among *Pterygodermatites* species including the nucleotide sequences of *P.* (*Me.*) *valladaresi* n. sp. obtained in this study (shown in bold). *Plectus aquatilis* was used as the outgroup.

A phylogenetic analysis based on the MT-CO1 gene sequences of *P.* (*Me.*) *valladaresi* n. sp. and other *Pterygodermatites* species available in GenBank was performed using the Neighbour-Joining distance method with the *p*-distance model [35] (Supplementary material Figure S1) and Maximum-Likelihood method with Tamura-Nei model [43] (Fig. 2), both with at least 1000 bootstrap replications in MEGA X [22]. The sequence of *Plectus aquatilis* Andrássy, 1985 (KX017524) was used as the outgroup.

## Results

### *Pterygodermatites* (*Mesopectines*) *valladaresi* n. sp., Table 1, Figures 3–9

ZooBank: urn:lsid:zoobank.org:act:90F17047-2D76-4654-99E6-90448F698974

**Table 1:** Morphological characteristics, host group and geographical distribution of *Pterygodermatites* (*Mesopectines*) species.

Species	Males			Females			Host group	Geographical distribution	References
	CP	Spicule length (right/left)	Fans	Prevulvar CP/Total CP	CP diff.	V			
*P.* (*Me.*) *alphi* (Lubimov, 1933)	68	Equal (100)	–	43/91	–	Ant	Primates: Calitrichidae, Cebidae, Cercopithecidae, Lemuridae	Russia	[13, 31, 41]
*P.* (*Me.*) *caucasica* (Schulz, 1927)	–	–	–	38–39/–	–	Ant	Rodentia: Muridae	Mongolia, Russia	[13, 44]
*P.* (*Me.*) *dollfusi* (Chabaud & Rousselot, 1956)	67–68	Equal (170)	2	48/87–91	Ant	Ant	Carnivora: Nandiniidae	Central African Republic	[8, 32]
*P.* (*Me.*) *fallax* (Jägerskiöld, 1904)	–	–	–	42/84	Post	Post	Rodentia: Sciuridae	Sumatra (Indonesia)	[20, 31]
*P.* (*Me.*) *harrisi* (Baylis, 1934)	62–67	Equal (75–85)	1–5	38–39/68–75	–	Ant–Post	Rodentia: Muridae	Tanzania	[4]
*P.* (*Me.*) *houdemeri* (Hsü, 1935)	65–66	Equal (230–245)	3–4	46–48/116–118	–	Post	Carnivora: Viverridae	Vietnam	[18]
*P.* (*Me.*) *kazachstanica* (Panine, 1956)	70	Equal (69)	–	45/71	–	–	Rodentia: Muridae, Sciuridae	Kazakhstan	[31]
*P.* (*Me.*) *leiperi* (Ortlepp, 1961)	62–66^1^	Equal	7	28–33/87–93^1^	Ant	Post	Carnivora: Viverridae	Haute-Volta (Burkina Faso), South Africa, Spain	[25]^1^, [26], [29]^2^, [31]^3^
	64–65^2^	(357–391)^1^		28–30/88–93^2^					
	64^3^	(360–366)^2^		29/87–90^3^					
		(350)^3^							
*P.* (*Me.*) *magna* (Kreis, 1937)	–	–	–	37/46	–	Post	Rodentia: Muridae	Angola	[13, 31]
*P.* (*Me.*) *mjoebergi* (Baylis, 1928)	65–66	Unequal	4–5	47–49/94–97^1^	Post	Post	Carnivora: Viverridae	China, Malaysia	[2]^1^, [9]^2^
		(118/319)		46/92^2^					
*P.* (*Me.*) *myonacis* (Ortlepp, 1961)	–	–	–	43–44/89–91	–	Post	Carnivora: Herpestidae	South Africa	[29]
*P.* (*Me.*) *niameyensis* Diouf, Diagne, Quilichini, Dobigny, Garba & Marchand, 2013	60–63	Subequal (55–70/65–80)	2-6	42–44/–	Ant	Ant	Rodentia: Muridae	Niger	[11]
*P.* (*Me.*) *nycticebi* (Mönnig, 1920)	54–70^1^	Equal	1^1^	43/93^1^	Vulva^1^	Junct	Primates: Callitrichidae, Cebidae, Daubentoniidae, Lorisidae, Galagidae, Pitheciidae	Germany, Japan, Java (Indonesia), Malaysia, Switzerland, USA	[19]^1^, [31], [33]^2^, [41]
	66^2^	(79–90)^1^	1–3^2^	30–42/68–94^2^	Post^2^				
		(80)^2^							
*P.* (*Me.*) *ortleppi* Quentin, 1969	75	Equal (50–67)	3	41/87–91	Vulva	Junct–Post	Rodentia: Muridae	Haute-Volta (Burkina Faso), Ivory Coast	[31]
*P.* (*Me.*) *paradoxuri* (Tubangui & Masiluñgan, 1937)	60–64	Subequal (220–260/212–255)	4	46–49/92	Ant	Post	Carnivora: Viverridae	Philippines	[46
*P.* (*Me.*) *quentini* Diouf, Quilichini, Granjon, Bâ & Marchand, 2013	58–61	Unequal (72–85/152–170)	2	40–42/68–71	Ant	Post	Rodentia: Muridae	Mali	[12]
*P.* (*Me.*) *ratti* (Khera, 1954)	56	Unequal (65/150)	5	40/57	Post	Ant	Rodentia: Muridae	India	[21]
*P.* (*Me.*) *senegalensis* Diouf, Bâ & Marchand, 2000	69–72	Equal (96–120)	1	40–43/90–94	Post	Ant	Rodentia: Muridae	Senegal	[10]
*P.* (*Me.*) *tani* (Hoeppli, 1929)	63–65^1^	Equal	3^1^	42–45/92–94^1^	Post	–	Rodentia: Muridae	China, Japan, Taiwan, Thailand	[16]^1^, [17]^2^, [41]
	64–68^2^	(62–78/68–76)^1^	2–3^2^						
		(76–81/70–84)^2^							
*P.* (*Me.*) *taterilli* (Baylis, 1928)	62–63	Unequal	4^1^	40–41/55–59?^1^	Post	Ant	Rodentia: Muridae	Ivory Coast, Haute-Volta (Burkina Faso), Nigeria	[3]^1^, [31]^2^
		(50/120)^1^	3–4^2^	41–42/64–74^2^					
		(40–67/110–135)^2^							
*P.* (*Me.*) *valladaresi* n. sp.	62–64	Unequal (62–90/123–139)	3–4	38–42/63–71	Post	Ant	Rodentia: Muridae	Canary Islands (Spain)	Present study
*P.* (*Me.*) *variabilis* Ghazi, Noor-Un-Nisa & Shafi, 1991	57–63	Unequal (76–77/1750–1760)	4	40–47/62–92	Vulva	Ant–Post	Rodentia: Muridae	Pakistan	[15]
*P.* (*Me.*) *vauceli* (Le van Hoa, 1965)	64	Unequal (65/150)	–	43–45/92	–	–	Rodentia: Sciuridae	Vietnam	[31]
*P.* (*Me.*) *whartoni* (Tubangui, 1931)	64^1^	Unequal	4^1,4^	42–43/89–93^3^	Post	Post	Rodentia: Muridae, Sciuridae	Japan, Papua New Guinea, Philippines, Taiwan	[16]^1^, [17]^2^, [31]^3^, [39]^4^, [41, 42, 45]
	60–62^2^	(75/155)^1^	3–4^2^	44–45/91–94^4^					
	62^3,4^	(65–83/138–170^2)^							
		(40/50)^3^?							
		(40/155)^4^							
*P.* (*Me.*) *wheeleri* (Sandground, 1933)	–	–	–	49–51/95–105	–	Post	Carnivora: Viverridae	Vietnam	[37]
*P.* (*Me.*) *witenbergi* Quentin & Wertheim, 1975	70–72	Subequal (72–85/80–84)	1–2	41–44/85–88	Post	Post	Rodentia: Muridae	Israel	[34]

All measurements are given in µm.

(CP) number of cuticular projection pairs; (CP diff.) Cuticular projections’ differentiation (combs to spines) in relation to the vulva; (V) position of the vulva in relation to the oesophagus–intestine junction; (Ant) anterior; (Junct) at junction; (Post) posterior; (Vulva) at vulvar level; (–) unknown data; (?) doubtful data. Superscript numbers in each row correspond to the reference for the different morphometric values reported for the same species.

Family Rictulariidae Hall, 1913

Genus *Pterygodermatites* Wedl, 1861

Subgenus *Pterygodermatites* (*Mesopectines*) Quentin, 1969

*Type host*: *Mus musculus* Linnaeus, 1758 (Rodentia: Muridae).

*Type locality*: La Oliva (28° 36′ 29.49″ N, 13° 55′ 54.64″ W) (Fuerteventura Island, Canary Archipelago, Spain) (Fig. 1).

*Other localities*: El Paso (28° 38′ 34.8″ N, 17° 51′ 3.2″ W) (La Palma Island, Canary Archipelago, Spain), Lagartario-Frontera (27° 46′ 29.9″ N, 17° 59′ 55.59″ W) (El Hierro Island, Canary Archipelago, Spain), Gasolinera-Antoncojo (28° 2′ 10.06″ N, 17° 12′ 52.02″ W) (La Gomera Island, Canary Archipelago, Spain), La Barrera (27° 59′ 45.35″ N, 15° 28′ 6.16″ W), Lomo del Conde (27° 58′ 11.68″ N, 15° 28′ 13.31″ W) and Pasadilla (27° 56′ 44.95″ N, 15° 28′ 15.97″ W) (Gran Canaria Island, Canary Archipelago, Spain) (Fig. 1).

*Site of infection*: small intestine.

*Prevalence*: overall prevalence in the Canary Archipelago was 2.2%; Fuerteventura Island (5.7%); Gran Canaria Island (12.2%); La Gomera Island (9.7%); La Palma Island (1.2%); and El Hierro Island (0.6%).

*Type* specimens: deposited in the Muséum National d’Histoire Naturelle, Paris [holotype, male No. 3, MNHN HEL1833; allotype, female No. 50, MNHN HEL1835; and 13 paratypes (1 male No. 1, MNHN HEL1834 and 12 females: No. 1, MNHN HEL1836, No. 13, MNHN HEL1837, No. 17, MNHN HEL1838, No. 22, MNHN HEL1839, No. 38, MNHN HEL1840, No. 39, MNHN HEL1841, No. 40, MNHN HEL1842, No. 41, MNHN HEL1843, No. 42, MNHN HEL1844, No. 46, MNHN HEL1845, No. 48, MNHN HEL1846, No. 49, MNHN HEL1847].

*Mitochondrial cytochrome c oxidase subunit I gene (MT-CO1) partial sequence (629 bp)*: GenBank, under accession no. OPO86098.

*Etymology:* the specific name of this nematode refers to the Spanish parasitologist Prof. Basilio Valladares from University of La Laguna and former head of the “Instituto Universitario de Enfermedades Tropicales y Salud Pública de Canarias”, a promoter of parasite biodiversity research in the Canary Islands.

#### Description

**Figure 3:**
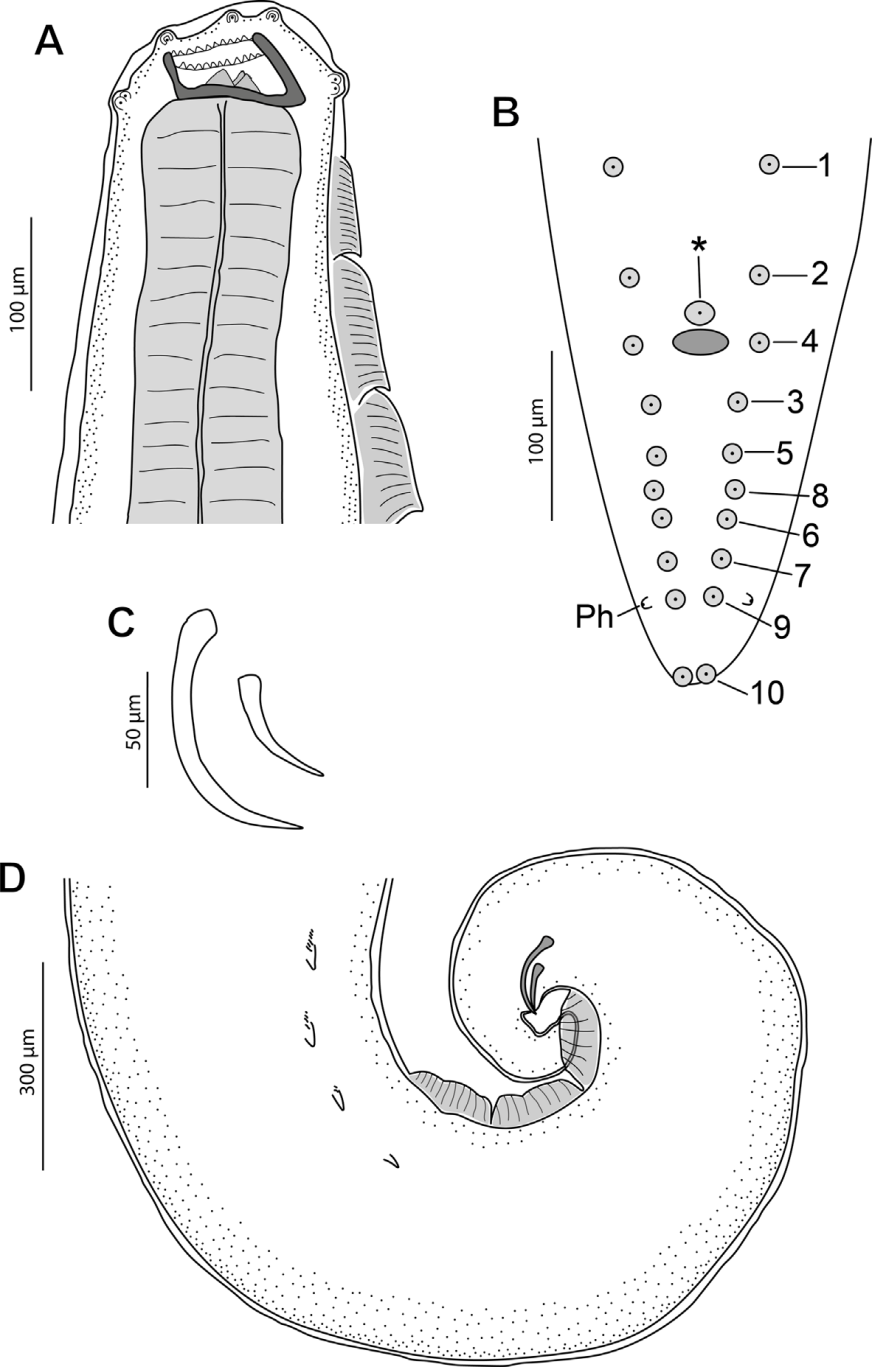
*Pterygodermatites* (*Mesopectines*) *valladaresi* n. sp., male. (A) Cephalic extremity, lateral view; (B) schematic drawing showing the arrangement of cloacal papillae; (C) detail of left and right spicules; (D) caudal extremity, lateral view. *, unpaired precloacal papilla; 1–10, pairs of cloacal papillae; Ph, phasmids.

**Figure 4:**
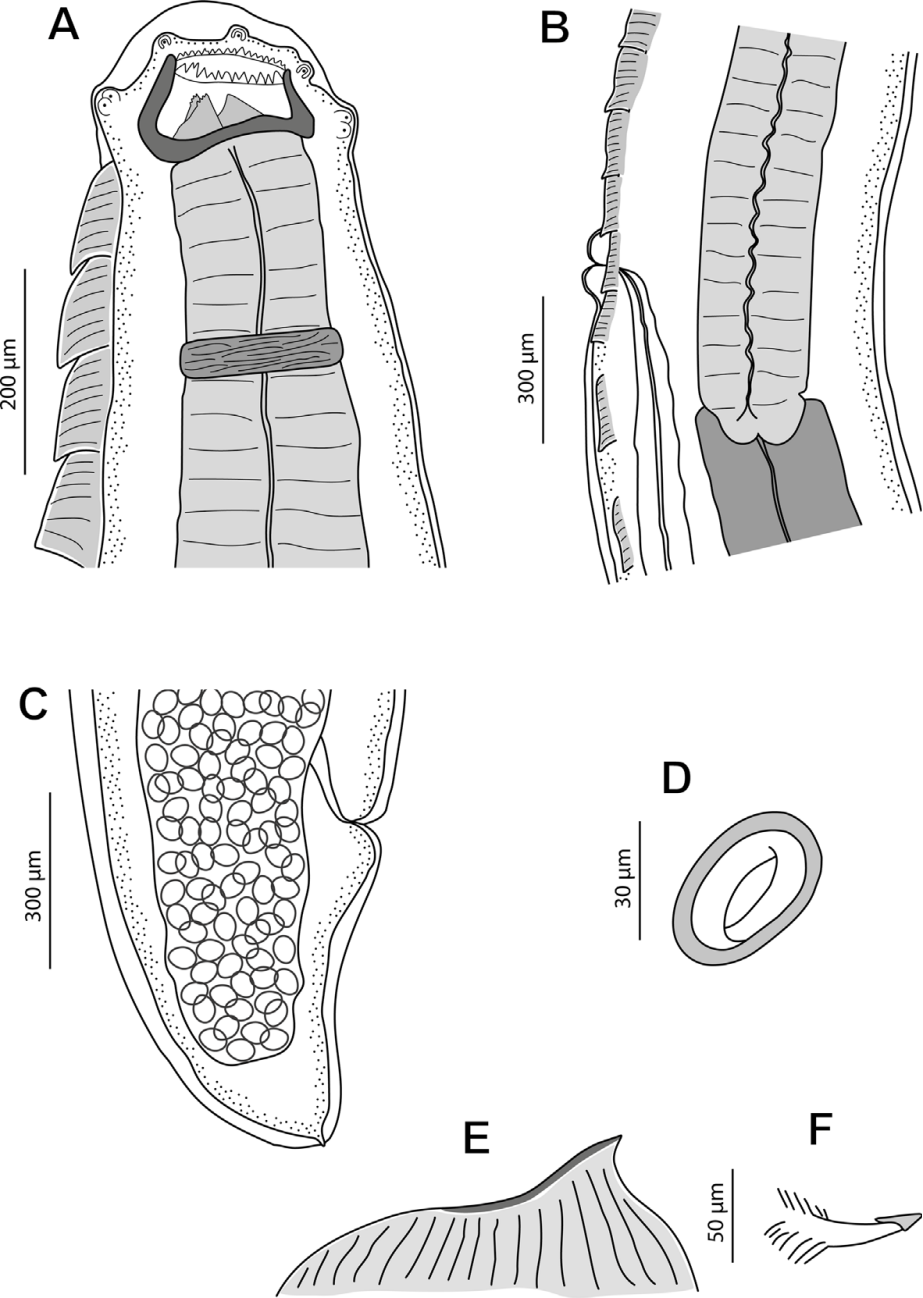
*Pterygodermatites* (*Mesopectines*) *valladaresi* n. sp., female. (A) Cephalic extremity, lateral view; (B) detail of vulvar region, lateral view; (C) tail, lateral view; (D) embryonated egg; (E) detail of a prevulvar cuticular comb (pair 21); (F) detail of a postvulvar cuticular spine (pair 56).

*General*: Medium-sized nematodes. Subapical oral opening, surrounded by an internal circle of 6 labial papillae (2 dorsal, 2 ventral and 2 lateral) and an external circle of 4 labial papillae plus 4 cephalic papillae arranged in 4 pairs (2 dorsal and 2 ventral) (Figs. 3A, 4A, 7D and 9E). Lateral amphids (Fig. 9E). Well-developed buccal capsule, dorsally oriented, with a more developed ventral wall in relation to the dorsal one (Figs. 3A and 4A). Oral opening surrounded by a crown of small regular denticles (Figs. 3A, 4A, 6A, 6C, 7D, 7E and 9E); three internal oesophageal teeth (1 dorsal and 2 lateroventral) at the bottom of the buccal capsule, of which only the lateroventral teeth are denticulated (Figs. 3A, 4A, 6C and 9E). Oesophagus with muscular and glandular portions. Two subventral rows of cuticular projections along the body, in form of combs and spines both in males and females (Figs. 3A, 4A, 4B, 4D, 4E, 6A, 6B, 7A–7C, 8A and 9A–9D).

**Figure 5:**
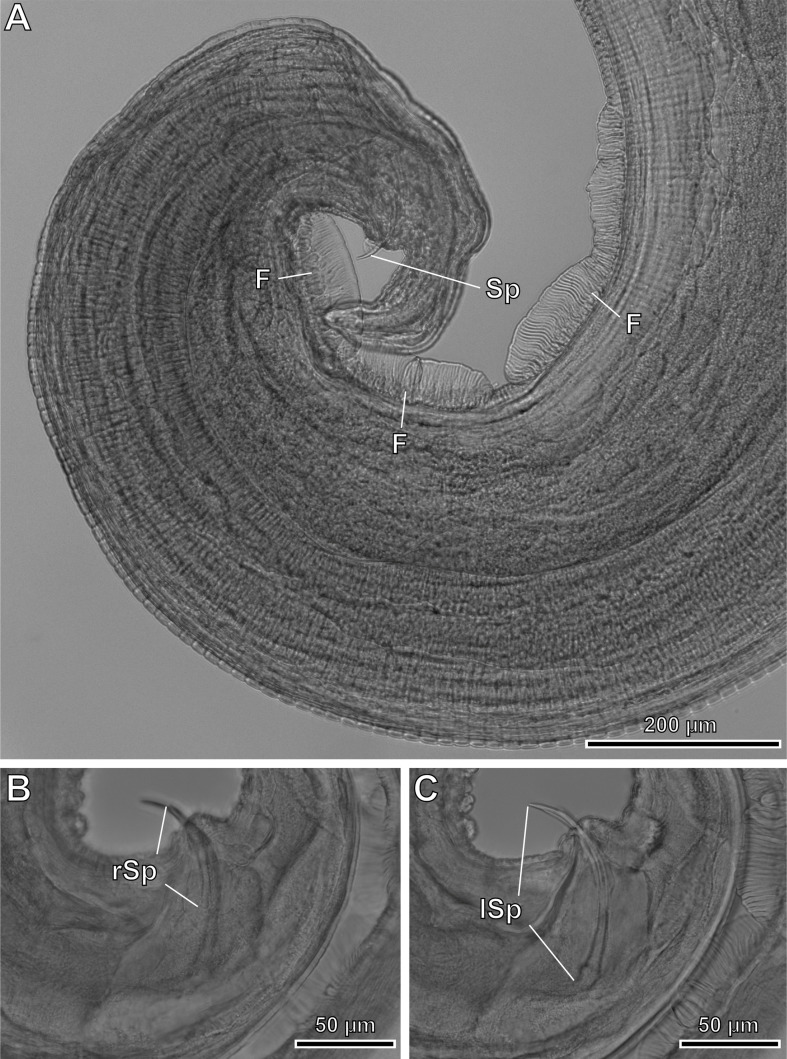
*Pterygodermatites* (*Mesopectines*) *valladaresi* n. sp., male, light microscopy. (A) Caudal extremity showing the presence of 3 midventral fans; (B) detail of right spicule; (C) detail of left spicule. F, fans; lSp, left spicule; rSp, right spicule; Sp, spicules.

**Figure 6:**
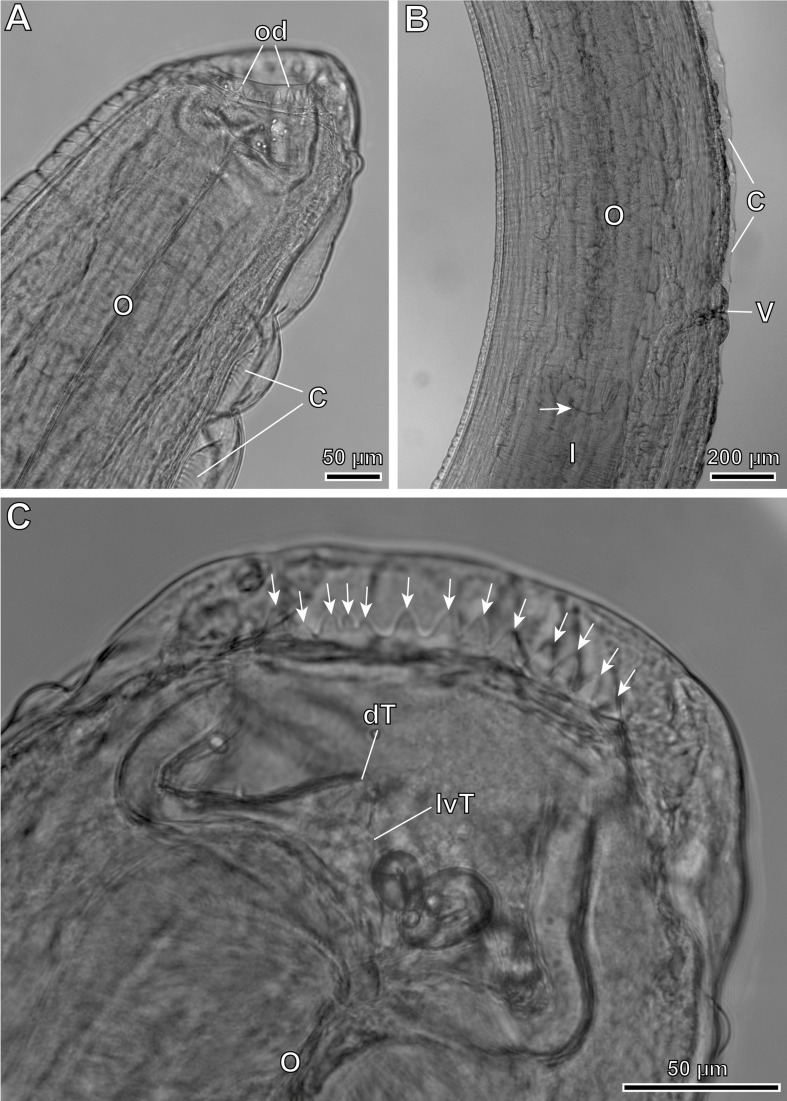
*Pterygodermatites* (*Mesopectines*) *valladaresi* n. sp., female, light microscopy. (A) Cephalic extremity, lateral view; (B) vulvar region showing the oesophagus-intestine junction (arrow); (C) buccal capsule showing the oral denticles present in the lateral side (arrows). C, cuticular combs; od, oral denticles; dT, dorsal oesophageal tooth; I, intestine; lvT, lateroventral oesophageal tooth; O, oesophagus; V, vulva.

**Figure 7:**
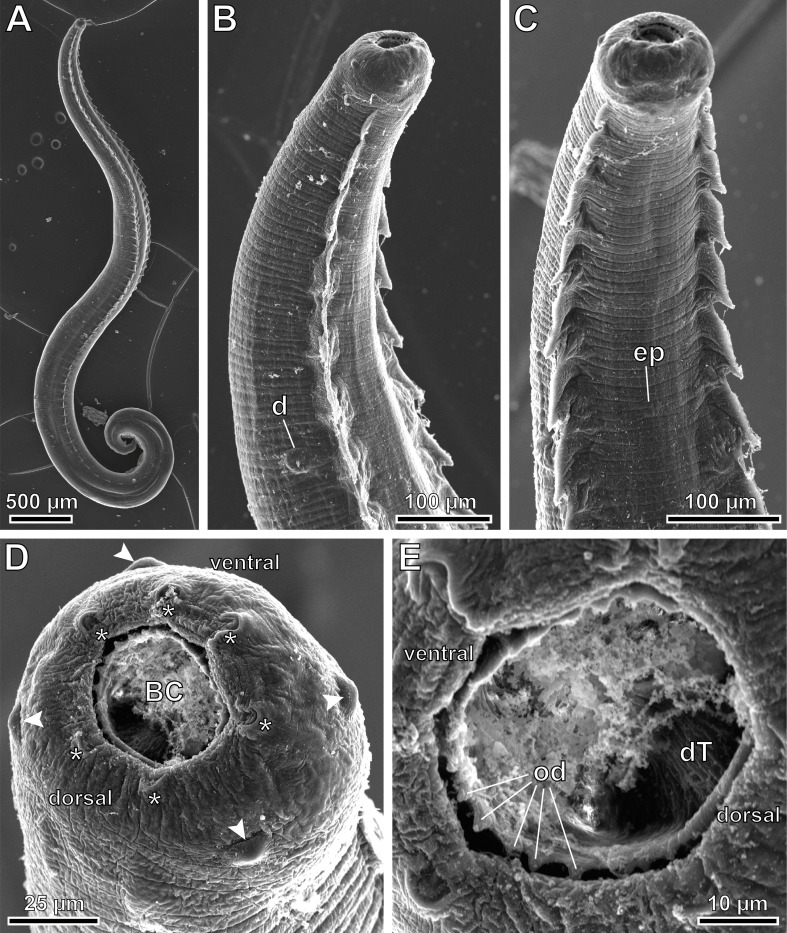
*Pterygodermatites* (*Mesopectines*) *valladaresi* n. sp., male, scanning electron microscopy. (A) General view; (B) cephalic extremity, lateral view; (C) cephalic extremity, ventral view; (D) apical view of the cephalic extremity showing labial and cephalic papillae; (E) detail of the buccal capsule showing the oral denticles. *, internal circle of 6 labial papillae; arrowhead, external circle of 4 labial papillae plus 4 cephalic papillae, grouped in pairs; BC, buccal capsule; d, deirid; dT, dorsal oesophageal tooth; ep, excretory pore; od, oral denticles.

**Figure 8:**
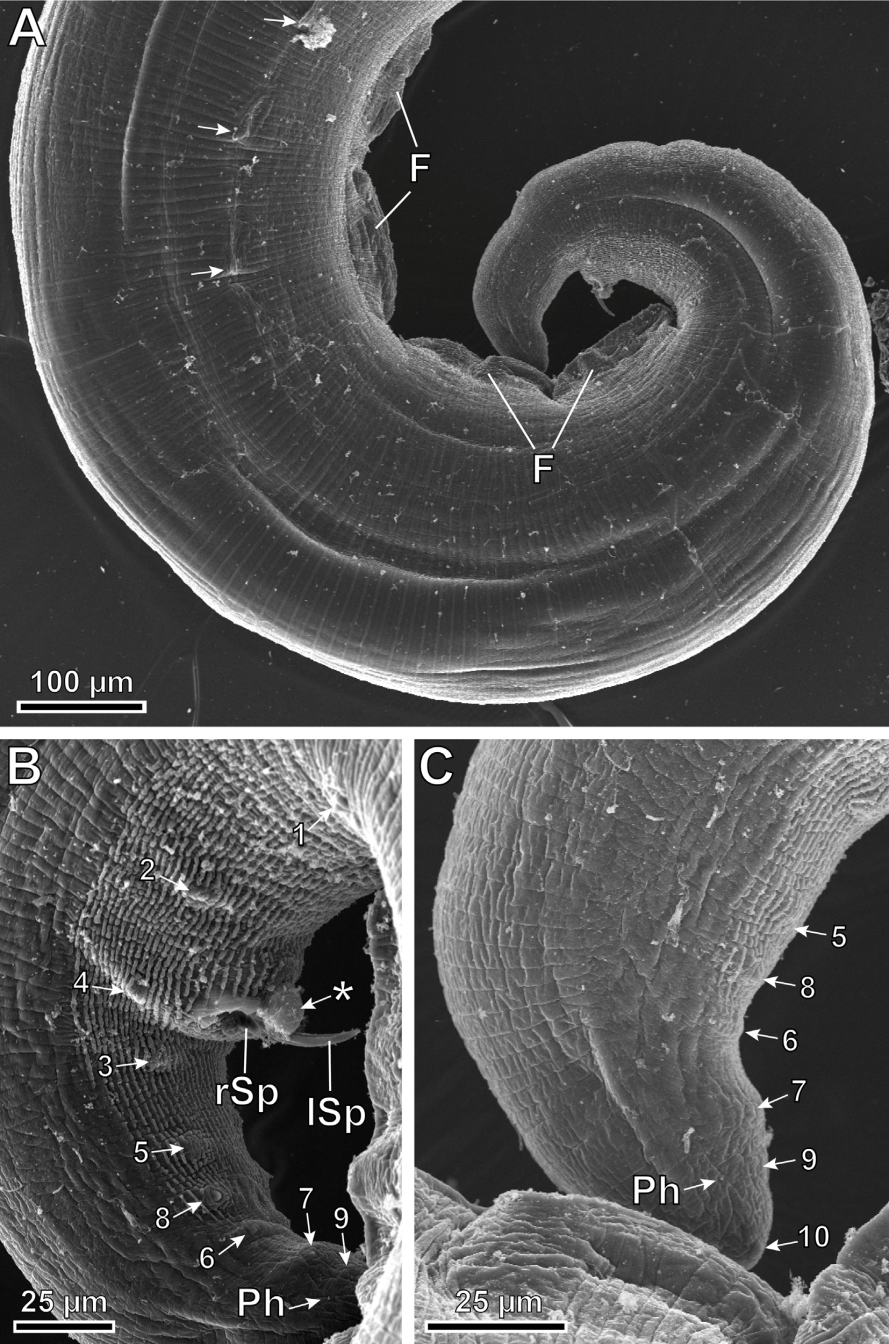
*Pterygodermatites* (*Mesopectines*) *valladaresi* n. sp., male, scanning electron microscopy. (A) Caudal extremity showing four midventral fans and the three last cuticular spines (arrows); (B) Detail of the cloacal region illustrating the location of papillae; (C) Detail of the posterior tip showing the location of papillae. *, unpaired precloacal papilla; 1–10, pairs of cloacal papillae; F, fans; lSp, left spicule; Ph, phasmids; rSp, right spicule.

**Figure 9:**
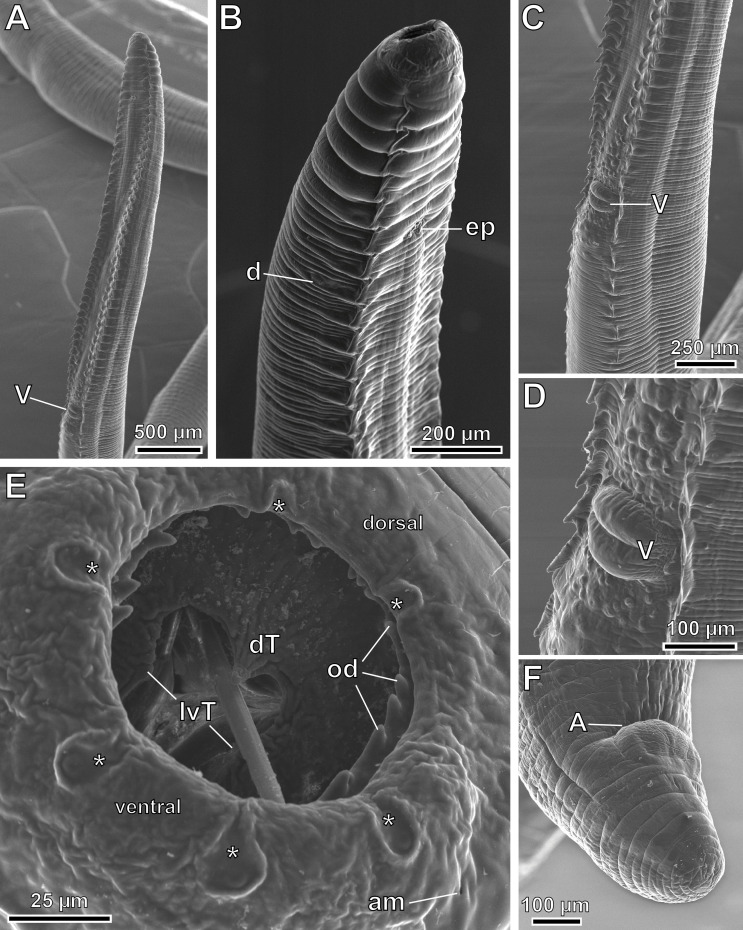
*Pterygodermatites* (*Mesopectines*) *valladaresi* n. sp., female, scanning electron microscopy. (A) General view of prevulvar region; (B) cephalic extremity, lateral view; (C) vulvar region; (D) detail of vulvar region showing the presence of numerous papillae; (E) detail of the oral opening; (F) posterior extremity. *, internal circle of 6 labial papillae; A, anus; am, amphid; d, deirid; dT, dorsal oesophageal tooth; ep, excretory pore; lvT, lateroventral oesophageal teeth; od, oral denticles; V, vulva.

*Male* (3 specimens; range, mean in parentheses, holotype measurements in brackets): Buccal capsule well developed and oriented dorsally (Figs. 3A and 7A–7C). Oral opening with a crown of 24 small regular denticles (Figs. 3A and 7D, 7E). Presence of three oesophageal teeth (Fig. 3A). Total pairs of combs and spines 62–64 [63]. Body length 3.86–8.04 mm (6.13 mm) [8.39 mm]; body width at the level of the oesophagus base 219–392 (331) [382]. Oesophagus length 1.42–2.41 mm (2.02 mm) [2.24 mm]; width at base 98–162 (125) [116]. Nerve ring located at 206–406 (306) [206] from the cephalic extremity, at the level of cuticular comb pairs 4–5. Deirids located at 525–753 (653) [753] from the cephalic extremity, at the level of cuticular comb pairs 8–9 (Fig. 7B). Excretory pore not measured; located at the level of cuticular comb pairs 6–7 (Fig. 7C). Posterior end of body strongly ventrally curved (Figs. 3D, 5A, 7A and 8A). Distance between the last cuticular spine and the tail tip 725–1376 (1033) [998] (Figs. 3D and 8A). Caudal extremity with 3–4 midventral fans anterior to the cloaca (Figs. 3D, 5A and 8A). Spicules unequal in size; right spicule 62–90 (73) [67]; left spicule 123–139 (131) [131] (Figs. 3C and 5B, 5C). Gubernaculum 18.0–20.6 (18.9) [20.6]. Total of 21 caudal papillae: 2 pairs of precloacal papillae, 1 unpaired precloacal papilla, 1 pair of pericloacal and 7 pairs of postcloacal papillae (Figs. 3B and 8B, 8C). Pairs of papillae aligned (Figs. 3B and 8B, 8C). Phasmids near the tail tip, laterally to the cloacal papillae 9 (Figs. 3B and 8B, 8C).

*Female* (13 gravid specimens; range, mean in parentheses, allotype measurements in brackets): Buccal capsule well developed and oriented dorsally (Figs. 4A, 6A, 6C and 9B). Oral opening presenting a crown of 19–26 small regular denticles (Figs. 4A, 6A, 6C and 9E). Presence of three oesophageal teeth (Figs. 4A and 9E). Body length 36.94–71.95 mm (57.79 mm) [54.78 mm]; body width at the level of the vulva 526–980 (719) [722]. Oesophagus length 4.02–6.55 mm (5.43 mm) [5.12 mm]; width at base 172–351 (233) [234]. Nerve ring located at 320–691 (487) [402] from the cephalic extremity, at the level of cuticular comb pairs 2–3. Deirids located at 908–1208 (1016) [1022] from the cephalic extremity, at the level of cuticular comb pairs 7–9 (Fig. 9B). Excretory pore not measured; located at the level of cuticular comb pair 7 (Fig. 9B). Total pairs of combs and spines 63–70 [67]; prevulvar pairs of combs 39–42 [42] (Fig. 4E); prevulvar combs very close together, becoming increasingly separated from each other after the vulva (Figs. 4B and 9C); postvulvar pairs of combs and spines 22–31 [25]; usually 6–8 postvulvar combs before its transformation into spines (Fig. 4F); transition from combs to spines posterior in relation to the vulva; distance between the last two spines around 2 mm. Vulva preequatorial with two prominent lips (Figs. 4B and 9C, 9D) and located at 4.05–6.76 mm (5.23 mm) [5.09 mm] from the cephalic extremity (Fig. 9A); anterior to the oesophagus-intestine junction, at a distance of 103–753 (391) [310] (Figs. 4B and 6B). Presence of numerous papillae located both before and after the vulva (Figs. 9C, 9D). Tail 464–764 (573) [526]; with a terminal spine (Figs. 4C and 9E). Eggs oval with a thick eggshell 43.7–48.9 × 33.4–36.0 (45.5 × 35.2) [48.9 × 33.4] (Fig. 4D).

#### Molecular analyses

The phylogenetic trees carried out with the Neighbour-Joining (Supplementary material Figure S1) and Maximum-Likelihood (Fig. 2) methods based on the MT-CO1 gene showed similar results. Pterygodermatites (*Me.*) *valladaresi* n. sp. is included in a clade together with two species of the subgenus *P.* (*Mesopectines*) [*P.* (*Me.*) *whartoni* (Tubangui, 1931) and *P.* (*Me.*) *nycticebi* (Mönnig, 1920)] with high bootstrap value (100%), and clearly separated from other clade including species of the subgenera *P.* (*Pterygodermatites*) and *P.* (*Paucipectines*), namely *P.* (*P.*) *plagiostoma* Wedl, 1861, *P.* (*Pa.*) *jaegerskioeldi* (Lent & Freitas, 1935) and *P.* (*Pa.*) *zygodontomis* (Quentin, 1967).

## Discussion

The new species *P.* (*Me.*) *valladaresi* n. sp. was included in the subgenus *P.* (*Mesopectines*) after an accurate analysis of the morphological characteristics of the buccal capsule and associated structures, and also of the male caudal extremity. The buccal capsule is oriented dorsally, with a more developed ventral wall in relation to the dorsal one. The oral opening is surrounded by a crown of regular denticles. There are three oesophageal teeth of similar development (one dorsal and two lateroventral). The lateroventral teeth are denticulated. Finally, the number, morphology and distribution of caudal papillae in males are other important features. They are arranged as follows: two precloacal pairs, one unpaired precloacal papilla, one pericloacal pair and seven postcloacal pairs. Moreover, they are sessile and aligned, and follow the Spirurida arrangement type of male cloacal papillae [7, 31]. Because of all these features, these rictulariids were attributed to the subgenus *P.* (*Mesopectines*) [6, 31].

In males, there are three characters useful for the specific discrimination between species: the number of cuticular projection pairs (combs and spines), the number of midventral fans, and the size of spicules [13, 31, 41]. Several species of the subgenus *P.* (*Mesopectines*) present the same number of cuticular projection pairs as the new species: *P.* (*Me.*) *harrisi* (Baylis, 1934), *P.* (*Me.*) *leiperi* (Ortlepp, 1961), *P.* (*Me.*) *niameyensis* Diouf, Diagne, Quilichini, Dobigny, Garba & Marchand, 2013, *P.* (*Me.*) *paradoxuri* (Tubangui & Masiluñgan, 1937), *P.* (*Me.*) *tani* (Hoeppli, 1929), *P.* (*Me.*) *taterilli* (Baylis, 1928), *P.* (*Me.*) *variabilis* Ghazi, Noor-Un-Nisa & Shafi, 1991, *P.* (*Me.*) *vauceli* (Le van Hoa, 1965) and *P.* (*Me.*) *whartoni* [3, 4, 11, 15–17, 29, 31, 39, 45, 46]. The unequal size of spicules differentiates the new species from *P.* (*Me.*) *harrisi*, *P.* (*Me.*) *leiperi*, *P.* (*Me.*) *niameyensis* and *P.* (*Me.*) *paradoxuri*; the size of the right spicule differentiates the new species from *P.* (*Me.*) *taterilli*; the size of the left spicule differentiates the new species from *P.* (*Me.*) *tani*, *P.* (*Me.*) *variabilis*, *P.* (*Me.*) *vauceli* and *P.* (*Me.*) *whartoni* (see Table 1). Males have not been studied in five species of the subgenus *P.* (*Mesopectines*), namely *P.* (*Me.*) *caucasica* (Schulz, 1927), *P.* (*Me.*) *fallax* (Jägerskiöld, 1904), *P.* (*Me.*) *magna* (Kreis, 1937), *P.* (*Me.*) *myonacis* (Ortlepp, 1961) and *P.* (*Me.*) *wheeleri* (Sandground, 1933) [8, 13, 20, 29, 31, 37]. These five species can be differentiated from *P.* (*Me.*) *valladaresi* n. sp. by some characteristics of female specimens, parasitized hosts and/or geographical distribution (see Table 1).

Another important criterion useful for species differentiation in male specimens is the number of midventral fans in the caudal extremity, located anteriorly to the cloaca. In *P.* (*Me.*) *valladaresi* n. sp., males have 3 or 4 midventral fans as in other species of the subgenus, namely *P.* (*Me.*) *houdemeri* (Hsü, 1935) [18], *P.* (*Me.*) *ortleppi* Quentin, 1969 [31], *P.* (*Me.*) *taterilli* [3], *P.* (*Me.*) *variabilis* [15] and *P.* (*Me.*) *whartoni* [16, 17, 39, 45]. However, male specimens of these species differ from *P.* (*Me.*) *valladaresi* n. sp. in the number of cuticular projection pairs and in the presence of equal-size spicules in the case of *P.* (*Me.*) *houdemeri* and *P.* (*Me.*) *ortleppi* [18, 31]; in the smaller size of the right spicule in *P.* (*Me.*) *taterilli* [3]; and in the size of the left spicule for *P.* (*Me.*) *variabilis* and *P.* (*Me.*) *whartoni* [15–17, 39, 45] (see Table 1).

Finally, with respect to spicules, 13 species have equal or subequal spicules: *P.* (*Me.*) *alphi* (Lubimov, 1933), *P.* (*Me.*) *dollfusi* (Chabaud & Rousselot, 1956), *P.* (*Me.*) *harrisi*, *P.* (*Me.*) *houdemeri*, *P.* (*Me.*) *kazachstanica* (Panine, 1956), *P.* (*Me.*) *leiperi*, *P.* (*Me.*) *niameyensis*, *P.* (*Me.*) *nycticebi*, *P.* (*Me.*) *ortleppi*, *P.* (*Me.*) *paradoxuri*, *P.* (*Me.*) *senegalensis* Diouf, Bâ & Marchand, 2000, *P.* (*Me.*) *tani* and *P.* (*Me.*) *witenbergi* Quentin & Wertheim, 1975 [4, 10, 11, 13, 16–18, 29, 31–34, 41, 46]. In the remaining species for which information on males is available, the spicules are unequal, including *P.* (*Me.*) *valladaresi* n. sp. Considering species with unequal spicules, the new species differs from *P.* (*Me.*) *mjoebergi* (Baylis, 1928) in the size of both right and left spicule and in number of midventral fans [9]; from *P.* (*Me.*) *quentini* Diouf, Quilichini, Granjon, Bâ & Marchand, 2013 and *P.* (*Me.*) *ratti* (Khera, 1954) in the size of the left spicule and number of midventral fans [12, 21]; from *P.* (*Me.*) *taterilli* in the size of the right spicule [3] and from *P.* (*Me.*) *variabilis*, *P.* (*Me.*) *vauceli* and *P.* (*Me.*) *whartoni* in the size of the left spicule [15–17, 31, 39, 45] (see Table 1).

Considering the caudal extremity of males, the arrangement of cloacal papillae is another particularly important feature to discriminate species between subgenera. The presence of ten pairs of cloacal papillae, plus an unpaired precloacal papilla and a pair of phasmids near the tail tip is a characteristic of all rictulariids [31]. However, cloacal papillae are difficult to observe and in several studies all the papillae have not been described [41]. Thus, male representatives of this nematode family have three types of posterior extremities according to the morphology and arrangement of cloacal papillae: the type Ascaridida, with a dorsolateral disposition of the pairs of papillae 1, 4 and 8; the type Spirurida, with a linear or almost linear arrangement of papillae; and a third type with grouped papillae and with the presence of some pedunculated papillae [7, 31]. The posterior extremity type Ascaridida is present in species of the genus *Rictularia* and in those of the subgenera *P.* (*Paucipectines*), *P.* (*Neopaucipectines*) and *P.* (*Pterygodermatites*); the posterior extremity type Spirurida is present in species of the subgenus *P.* (*Mesopectines*) and the third type of posterior extremity with grouped and pedunculated papillae is present in representatives of the subgenus *P.* (*Multipectines*) [5, 6, 27, 31].

In females, the most discriminant characters between species are: the prevulvar and total number of cuticular projection pairs, the body level where the transition from combs to spines occurs and the position of the vulva in relation to the oesophagus-intestine junction [13, 31, 41]. The number of prevulvar cuticular projection pairs in *P.* (*Me.*) *valladaresi* n. sp. is similar to that found in most species of the subgenus *P.* (*Mesopectines*). Considering prevulvar comb pairs, the new species differs from *P.* (*Me.*) *dollfusi*, *P.* (*Me.*) *houdemeri*, *P.* (*Me.*) *kazachstanica*, *P.* (*Me.*) *mjoebergi*, *P.* (*Me.*) *paradoxuri*, *P.* (*Me.*) *vauceli* and *P.* (*Me.*) *wheeleri*, which have a higher number of prevulvar comb pairs [2, 8, 18, 31, 37, 46] (see Table 1). It also differs from *P.* (*Me.*) *leiperi*, which has a small number of prevulvar comb pairs [29, 31]. With respect to the total number of cuticular projection pairs, *P.* (*Me.*) *valladaresi* n. sp. has a much lower number of pairs than *P.* (*Me.*) *alphi*, *P.* (*Me.*) *dollfusi*, *P.* (*Me.*) *fallax*, *P.* (*Me.*) *houdemeri*, *P.* (*Me.*) *leiperi*, *P.* (*Me.*) *mjoebergi*, *P.* (*Me.*) *myonacis*, *P.* (*Me.*) *nycticebi*, *P.* (*Me.*) *ortleppi*, *P.* (*Me.*) *paradoxuri*, *P.* (*Me.*) *senegalensis*, *P.* (*Me.*) *tani*, *P.* (*Me.*) *variabilis*, *P.* (*Me.*) *vauceli*, *P.* (*Me.*) *whartoni*, *P.* (*Me.*) *wheeleri* and *P.* (*Me.*) *witenbergi* [2, 8, 10, 15–20, 29, 31–34, 37, 39, 45, 46] (see Table 1). Contrarily, the new species has a higher number of cuticular projection pairs than *P.* (*Me.*) *magna* and *P.* (*Me.*) *ratti* [13, 21, 31] (see Table 1). Thus, considering the prevulvar and total cuticular projection pairs of females, *P.* (*Me.*) *harrisi*, *P.* (*Me.*) *niameyensis*, *P.* (*Me.*) *quentini* and *P.* (*Me.*) *taterilli* [3, 4, 11, 12, 31] are the most similar species to *P.* (*Me.*) *valladaresi* n. sp. In both *P.* (*Me.*) *niameyensis* and *P.* (*Me.*) *quentini*, the differentiation of combs into spines is anterior in relation to the position of the vulva. Differences between both *P.* (*Me.*) *harrisi* and *P.* (*Me.*) *taterilli* and the new species must be established by comparing male specimens, in particular the size of spicules (see Table 1).

In *P.* (*Me.*) *valladaresi* n. sp., the differentiation of combs into spines is clearly posterior to the vulva. In fact, there are 6 to 8 postvulvar pairs of combs before their transformation into spines. A postvulvar differentiation of cuticular projections (combs into spines) is only found in nine species of this subgenus, namely *P.* (*Me.*) *fallax*, *P.* (*Me.*) *mjoebergi*, *P.* (*Me.*) *nycticebi*, *P.* (*Me.*) *ratti*, *P.* (*Me.*) *senegalensis*, *P.* (*Me.*) *tani*, *P.* (*Me.*) *taterilli*, *P.* (*Me.*) *whartoni* and *P.* (*Me.*) *witenbergi* [2, 3, 10, 16, 17, 19–21, 31, 33, 34, 39, 45]. All these species have already been differentiated from *P.* (*Me.*) *valladaresi* n. sp. in the previous paragraph. The remaining species of the subgenus *P.* (*Mesopectines*) have a prevulvar, vulvar or unknown level of transition of combs to spines (see Table 1).

The position of the vulva in relation to the oesophagus-intestine junction is another important female character, which is useful to differentiate *P.* (*Me.*) *valladaresi* n. sp. from the remaining species of the subgenus *P.* (*Mesopectines*). Although there may be some intraspecific variability, the position of the vulva in relation to the oesophagus-intestine junction is, in general, a constant character for species [30]. In the new species, the position of the vulva is anterior to the oesophagus-intestine junction. A similar position is found in nine species, namely *P.* (*Me.*) *alphi*, *P.* (*Me.*) *caucasica*, *P.* (*Me.*) *dollfusi*, *P.* (*Me.*) *harrisi*, *P.* (*Me.*) *niameyensis*, *P.* (*Me.*) *ratti*, *P.* (*Me.*) *senegalensis*, *P.* (*Me.*) *taterilli* and *P.* (*Me.*) *variabilis* [3, 4, 7, 9, 10, 12, 14, 20, 30, 43]. In this case, using the previously discussed characters, all these species were already differentiated from the new species, except for *P.* (*Me.*) *caucasica*. The available data of *P.* (*Me.*) *caucasica* are very scarce and concern only females [13, 44]; however, the number of prevulvar cuticular projection pairs is slightly lower than in the new species. Additionally, the parasitized host (*Meriones meridianus*) and the geographical distribution (Mongolia and North Caucasus, Russia) are other criteria to distinguish *P.* (*Me.*) *caucasica* from the new species. The remaining species of the subgenus *P.* (*Mesopectines*) present a posterior or unknown position of the vulva in relation to the oesophagus-intestine junction (see Table 1).

The geographical distribution and host range are other aspects considered in the present study. Species of *P.* (*Mesopectines*) are parasites of Rodentia, Carnivora and Primates and have been found in Asia and Africa, except for some species parasites of Primates detected in zoos, e.g. *P.* (*Me.*) *nycticebi* in zoos of Chicago, Oklahoma, Washington or Wisconsin (USA) [14, 28, 47, 48], Germany [24, 40], Japan [19, 38] or Switzerland [1]. *Pterygodermatites* (*Me.*) *valladaresi* n. sp. was found on five islands of the Canary Archipelago parasitizing *Mus musculus* (Fig. 1). In the case of *P.* (*Mesopectines*) species parasitizing rodents, numerous individuals have been collected from rodents of the families Muridae and Sciuridae (see Table 1). In Muridae, other than *P.* (*Me.*) *valladaresi* n. sp., only two species have been described parasitizing species of the genus *Mus*, namely *P.* (*Me.*) *magna* in *Mus* sp. from Angola and *P.* (*Me.*) *witenbergi* in *Mus musculus* from Israel [13, 31, 34]. In the Canary Archipelago, there are only three species of murids: *M. musculus*, *R. norvegicus* and *R. rattus*. During the present study, between the years 2008 and 2012, we found specimens of another *Pterygodermatites* species in three *R. rattus* trapped in the same biotopes of Fuerteventura Island (La Oliva) and El Hierro Island (Lagartario) [36]. Among other aspects, the *Pterygodermatites* individuals found in *R. rattus* were characterized by having 65–66 cuticular projection pairs, subequal spicules (right: 62–64 µm; left: 69–72 µm) and 2 or 3 midventral fans (unpublished data). Thus, *P.* (*Me.*) *valladaresi* n. sp. may be specific to *M. musculus*.

Quentin [31] argued about the geographic origin and evolution of rictulariids. For the genus *Pterygodermatites*, it seems that its geographic origin was an area of the Palearctic region between Siberia and Canada, and these archaic species belong to the subgenus *P.* (*Paucipectines*). Thus, this subgenus includes the most primitive species within the genus *Pterygodermatites*, which are parasites of Cricetidae rodents in the Palearctic and Nearctic regions. In fact, Quentin [31] considered the apical oral opening, the reduced number of prevulvar combs and the type Ascaridida for the arrangement of cloacal papillae as the primitive characters present in the subgenus *P.* (*Paucipectines*). According to this author, the remaining subgenera, including *P.* (*Mesopectines*), would have evolved from this archaic subgenus: the buccal capsule became progressively dorsal, oral ornamentations became increasingly diverse and cloacal papillae became more or less aligned and grouped. For the subgenus *P.* (*Mesopectines*), the buccal capsule is more or less dorsal according to the species and the oral ornamentations are variable, with species showing a crown of regular denticles as occurs in *P.* (*Me.*) *valladaresi* n. sp., while other species show quadrangular denticles [e.g. *P.* (*Me.*) *whartoni*] or with two ventral apophysis [e.g. *P.* (*Me.*) *leiperi*].

In conclusion, the morphologic characteristics of the buccal capsule and associated structures, the particular characters of males (arrangement of cloacal papillae, number of cuticular projection pairs, size of spicules, and number of fans) and females (number of prevulvar and total cuticular projection pairs, body level of the transition from combs to spines and position of the vulva in relation to the oesophagus-intestine junction), the molecular data, and the geographical distribution and parasitized host identify the discovered nematode as a new species of the subgenus *P.* (*Mesopectines*).

## Conflict of interest

The authors declare that they have no conflict of interest.

## Supplementary material

The supplementary material of this article is available at https://www.parasite-journal.org/10.1051/parasite/2022057/olm.**Figure S1:**
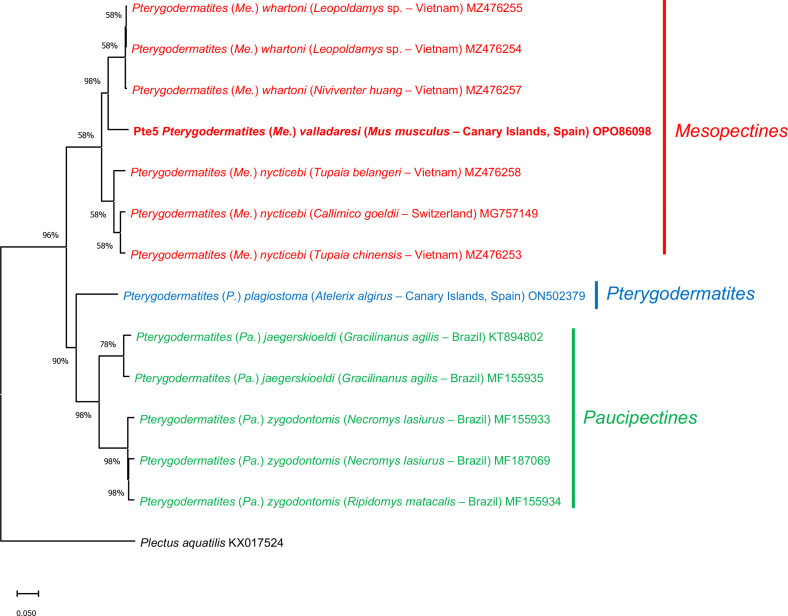
Phylogenetic analysis, based on the Neighbour-Joining method, of *Pterygodermatites* species based on the MT-CO1 gene sequences. *Plectus aquatilis* was used as the outgroup.
